# Nintedanib decreases muscle fibrosis and improves muscle function in a murine model of dystrophinopathy

**DOI:** 10.1038/s41419-018-0792-6

**Published:** 2018-07-10

**Authors:** Patricia Piñol-Jurado, Xavier Suárez-Calvet, Esther Fernández-Simón, Eduard Gallardo, Natalia de la Oliva, Anna Martínez-Muriana, Pedro Gómez-Gálvez, Luis M. Escudero, María Pérez-Peiró, Lutz Wollin, Noemi de Luna, Xavier Navarro, Isabel Illa, Jordi Díaz-Manera

**Affiliations:** 1 Neuromuscular disorders Unit, Neurology department, Universitat Autònoma de Barcelona, Hospital de la Santa Creu I Sant Pau, Barcelona, Spain; 2 Centro de Investigación Básica en Red en Enfermedades Raras (CIBERER), Madrid, Spain; 3 Department of Cell Biology, Physiology and Immunology, Institute of Neurosciences, Universitat Autònoma de Barcelona, Bellaterra, Barcelona, Spain; 4 Centro de Investigación Biomédica en Red sobre Enfermedades Neurodegenerativas (CIBERNED), Valencia, Spain; 5 Departamento de Biología Celular, Universidad de Sevilla and Instituto de Biomedicina de Sevilla (IBiS), Hospital Universitario Virgen del Rocío/CSIC/Universidad de Sevilla, 41013 Sevilla, Spain; 6 Immunology & Respiratory Diseases Research, Boehringer-Ingelheim, Biberach, Germany

## Abstract

Duchenne muscle dystrophy (DMD) is a genetic disorder characterized by progressive skeletal muscle weakness. Dystrophin deficiency induces instability of the sarcolemma during muscle contraction that leads to muscle necrosis and replacement of muscle by fibro-adipose tissue. Several therapies have been developed to counteract the fibrotic process. We report the effects of nintedanib, a tyrosine kinase inhibitor, in the *mdx* murine model of DMD. Nintedanib reduced proliferation and migration of human fibroblasts in vitro and decreased the expression of fibrotic genes such as *COL1A1*, *COL3A1*, *FN1*, *TGFB1,* and *PDGFA*. We treated seven *mdx* mice with 60 mg/kg/day nintedanib for 1 month. Electrophysiological studies showed an increase in the amplitude of the motor action potentials and an improvement of the morphology of motor unit potentials in the animals treated. Histological studies demonstrated a significant reduction of the fibrotic areas present in the skeletal muscles. Analysis of mRNA expression from muscles of treated mice showed a reduction in *Col1a1*, *Col3a1*, *Tgfb1*, and *Pdgfa*. Western blot showed a reduction in the expression of collagen I in skeletal muscles. In conclusion, nintedanib reduced the fibrotic process in a murine model of dystrophinopathy after 1 month of treatment, suggesting its potential use as a therapeutic drug in DMD patients.

## Introduction

Duchenne muscle dystrophy (DMD) is a genetic disorder produced by mutations in the *dystrophin* gene. DMD patients develop muscle weakness that usually starts at an age of 5–7 years and progresses quickly. At 14 years of age, most of the patients have lost ambulation. At 20 years, patients are completely dependent on care givers or relatives owing to severe muscle weakness. Respiratory muscle failure and/or myocardiopathy are the main causes of mortality in these patients^1,2^.

Several therapeutic strategies have been tested in murine models of DMD, and some of them have also been studied in clinical trials in patients^3^. Cell therapy using different stem cells, such as myoblasts or mesoangioblasts, have been tested both in animals and in patients^4^. Although results of animal experiments have shown promising results, tests in humans have not lead to any functional change in the patients treated^5,6^. Gene-based strategies, such as exon-skipping or readthrough of null mutations have demonstrated to restore the expression of dystrophin in muscles fibers both in mice and in patients^7,8^. Ataluren is at present commercialized in Europe to treat patients with non-sense mutations, and eteplirsen is being commercialized in USA to treat DMD patients with skipping of the exon 51^9,10^. CRISPR-Cas9 is a promising genetic strategy, that could be useful in patients although it has only been tested in animal models and cell cultures so far^11,12^. Drugs interfering the process of muscle degeneration are another potential strategy to treat muscle dystrophies. The process of muscle degeneration in DMD patients has been thoroughly studied. The absence of dystrophin weakens muscle membrane, leading to contraction-induced muscle fiber damage and death^13^. Muscle fiber loss is associated to expansion of fibro-adipose tissue producing muscle weakness^14^. Several cytokines and growth factors have been related to skeletal muscle fibrosis, although it has been reported that transforming growth factor β (TGF-β) is the most important factor in this process^15–17^. Several therapeutic strategies trying to decrease TGF-β activity have been developed, which have been shown to reduce fibrous tissue but also to increase inflammatory infiltration^18,19^. These results have promoted the investigation of other growth factors involved in muscle fibrosis.

Platelet-derived growth factors (PDGFs) are associated with multiple cellular processes such as proliferation, migration, and cell differentiation^20^. PDGF have been implicated in a broad range of diseases such as cancer, atherosclerosis and fibrosis. Several evidences supporting a role on muscle fibrosis of PDGF-AA have been published justifying therapeutic interventions targeting the PDGF signaling cascade^21,22^. Nintedanib is a tyrosine kinase inhibitor (TKI) also targeting fibroblast growth factor receptor (FGFR) 1 and 2, PDGF receptors α and β and vascular endothelial growth factor receptor (VEGFR)^23^. Nintedanib is approved for the treatment of patients with idiopathic pulmonary fibrosis (IPF), a condition in which expansion of the fibrotic tissue is crucial^24,25^. The anti-fibrotic activity of nintedanib has been confirmed in primary lung fibroblasts from patients with IPF and in dermal fibroblasts from patients with systemic sclerosis^25,26^.

In this study, we explored the effect of nintedanib on human fibroblasts obtained from muscle biopsies and on muscle fibrosis and function in the *mdx* mouse model of DMD.

## Results

### Nintedanib reduced proliferation, migration, and mRNA expression of fibrotic markers in human fibroblasts

**Fig. 1:**
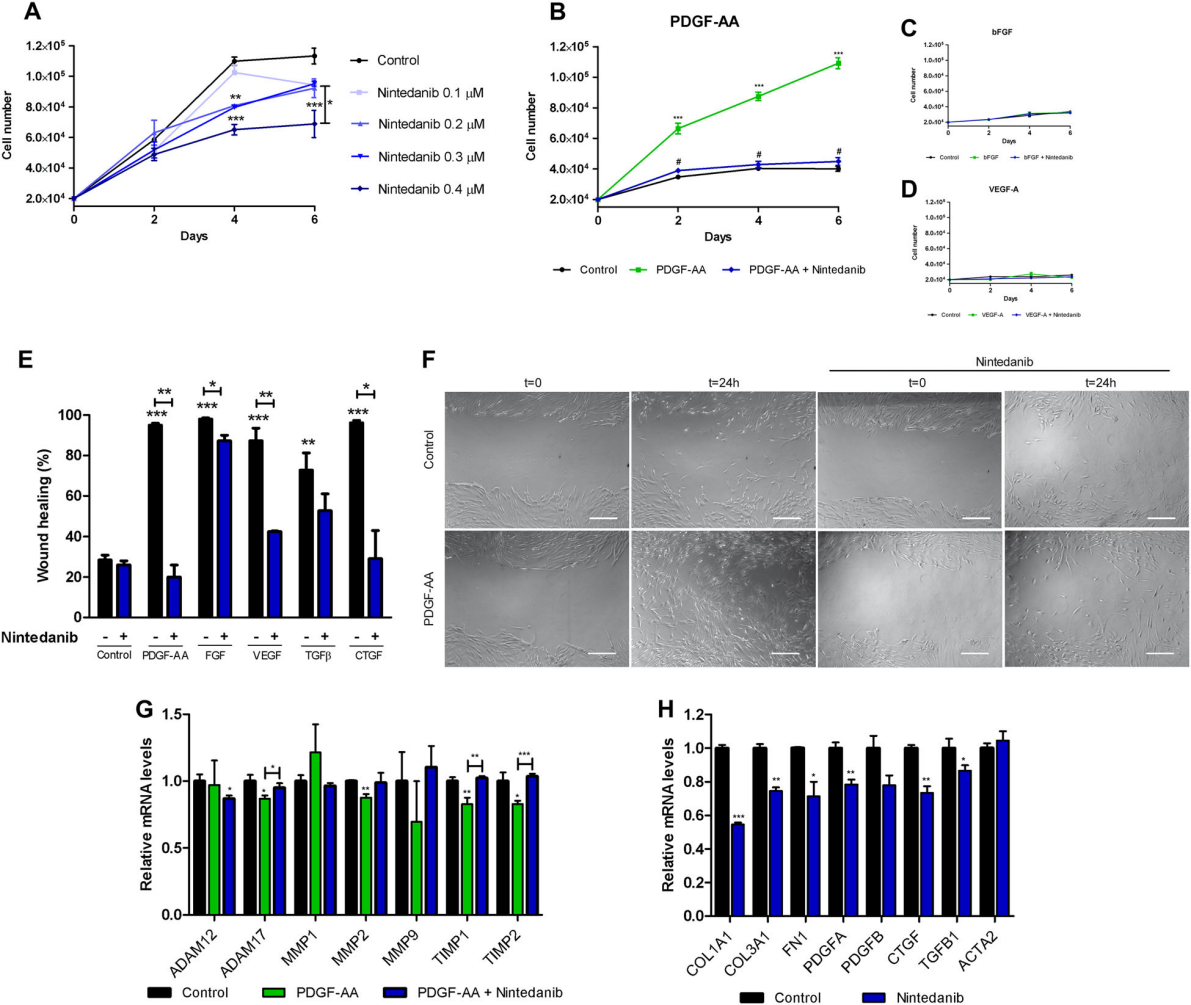
Effect of nintedanib on fibroblasts from healthy human muscle in vitro. Fibroblasts proliferation was analyzed after 2, 4, and 6 days of culture (**a–d**). We cultured fibroblast with increasing concentrations of nintedanib in a culture containing 10% fetal bovine serum (FBS) and we observed decreased fibroblast proliferation after 4 and 6 days in a dose-dependent manner (**a**). Fibroblasts were cultured in the presence of platelet-derived growth factor AA (PDGF-AA, 10 ng/mL) (**b**), basic fibroblast growth factor (FGFb, 10 ng/mL),(**c)** or vascular endothelial growth factor A (VEGF-A, 50 ng/mL) (**d**) with 1% FBS with or without nintedanib 0.4 μM. Only PDGF-AA increased cell proliferation, an effect that was reversed with the addition of nintedanib to the culture. Fibroblast migration was analyzed using a scratch assay. Nintedanid treatment at 0.4 μM reverted the promigratory effect of PDGF-AA, FGFb, VEGF, and CTGF (**e**). Representative images of this assay are shown in (**f**). Nintedanib treatment at 0.4 μM (blue bars) reverted the effect of PDGF-AA in the expression of ADAM metallopeptidase domain 17 (*ADAM-17)*, tissue inhibitor of metalloproteinase 1 *(TIMP-1)* and tissue inhibitor of metalloproteinase 2 *(TIMP-2)* (**g**). Nintedanib treatment at 0.4 μM (blue bars) produced a statistically significant reduction of collagen type I alpha 1 chain *(COL1A1)*, collagen type III alpha 1 chain *(COL3A1)*, fibronectin 1 (*FN1)*, platelet-derived growth factor A *(PDGFA)*, connective tissue growth factor *(CTGF)*, transforming growth factor beta 1 *(TGFβ1)* expression compared with control samples (black bars) analyzed by qPCR (**h**). Data are expressed as means ± SD. *N* = 3 per group. *P < 0.05, **P < 0.01, ***P < 0.005

In muscle dystrophies, activated fibroblasts proliferate and express high levels of extracellular proteins leading to the expansion of fibrotic tissue^27,28^. We analyzed whether nintedanib was able to decrease fibroblast proliferation in vitro. Nintedanib significantly reduced fibroblast proliferation in a dose-dependent manner reaching the highest effect using a concentration of 0.4 μM (Fig. 1a) without any cytotoxic effect measured by cell viability and apoptosis assays (Fig. S1A, B). Accordingly, the expression of the gene PT53 (tumor protein p53) and the number of Ki-67+ cells, which are well known cell cycle markers, were significantly lower in nintedanib-treated cells than in control non-treated cells (Fig. S1C, D). To study which growth factor signaling cascade targeted by nintedanib affects more prominently fibroblasts proliferation, we cultured human fibroblasts in the presence of recombinant PDGF-AA, recombinant human basic fibroblast growth factor (bFGF), or recombinant human vascular endothelial growth factor (VEGF)-A, with or without nintedanib (Fig. 1b–d). In order to avoid possible interferences with other growth factors present in the fetal bovine serum (FBS), we cultured the cells with 1% FBS only. PDGF-AA significantly increased fibroblast proliferation at days 2, 4, and 6 (Fig. 1b) but not bFGF or VEGF (Fig. 1c, d). This increase was completely blocked by the addition of nintedanib at 0.4 μM (Fig. 1b).

After muscle damage, activated fibroblasts gain migratory capabilities that allow them to move to the site of injury^29,30^. Using a scratch assay, we observed that nintedanib blocked cell migration promoted by PDGF-AA, bFGF, VEGF, and collagen tissue growth factor (CTGF) (Fig. 1e). The highest effect of nintedanib on cell migration was observed when PDGF-AA was added to the culture (Fig. 1e, f) and it was dose dependent (Fig. S2A, B). Transwell experiments confirmed these results showing that nintedanib reduced the effect of PDGF-AA in migration (Fig. S2C). Furthermore, nintedanib reverted significantly the effect of PDGF-AA on expression of the genes related with cell migration such as *ADAM-17 (*ADAM metallopeptidase domain 17), *TIMP-1* (tissue inhibitor of metalloproteinase 1), and *TIMP-2* (tissue inhibitor of metalloproteinase 2) (Fig. 1g). No significant differences were found in *ADAM-12 (*ADAM metallopeptidase domain 12), *MMP1* (metalloproteinase 1), *MMP2* (metalloproteinase 2), and *MMP9* (metalloproteinase 9) (Fig. 1g). To know whether nintedanib treatment modified mRNA expression of skeletal muscle fibroblasts in vitro we analyzed the expression of genes related with muscle fibrosis using Real-Time PCR after 4 days of culture. We observed that nintedanib at 0.4 μM reduced significantly the expression of *COL1A1* (Collagen type I alpha 1 chain), *COL3A1* (collagen type III alpha 1 chain), *FN1* (Fibronectin 1), *CTGF* (connective tissue growth factor)*, PDGFA* (platelet-derived growth factor A), and *TGFB1* (transforming growth factor beta 1) (Fig. 1h). In contrast, we did not observe significant differences in the expression of *ACTA-2* (actin, alpha 2, smooth muscle, aorta) or *PDGF-B* (platelet-derived growth factor B).

### Nintedanib had no effect on myoblast differentiation to myotubes in vitro

To analyze whether nintedanib could interfere with myoblast differentiation we cultured confluent human myoblasts in differentiation medium with increasing doses of nintedanib for 7 days. No differences in differentiation index between control myotubes and myotubes treated with nintedanib were observed (Fig. S3A). In addition, we did not find differences in the differentiation index of myoblasts cultured in a medium containing PDGF-AA, b-FGF, or VEGF-A with or without nintedanib (Fig. S3B, C). We also studied whether myotubes treated with nintedanib were more resistant to chemical damage using 0.25 mM sodium dodecyl sulfate (SDS), and we did not see an effect of nintedanib on sarcolemma repair (Figure S3D).

### Nintedanib treatment improves electrophysiological tests in mdx mice

We treated 10 months old *mdx* mice (*n* = 7) with nintedanib (from now on nintedanib-treated *mdx* mice) at a dose of 60 mg/kg/day orally for 1 month and compared muscle function tests with 10 months old non-treated *mdx* mice (from now on *mdx* mice; *n* = 5) and 10 months old C57 wild-type mice (from now on WT mice; *n* = 5). We first performed a DigiGait analysis, which allows identifying differences in the walking pattern of healthy and dystrophic mice. We did not find differences between WT mice, *mdx* mice, and nintedanib-treated *mdx* mice, suggesting that the severity of weakness involving muscles of the paws in this murine model at this age is not enough to find differences using this technique (Fig S4).

**Fig. 2:**
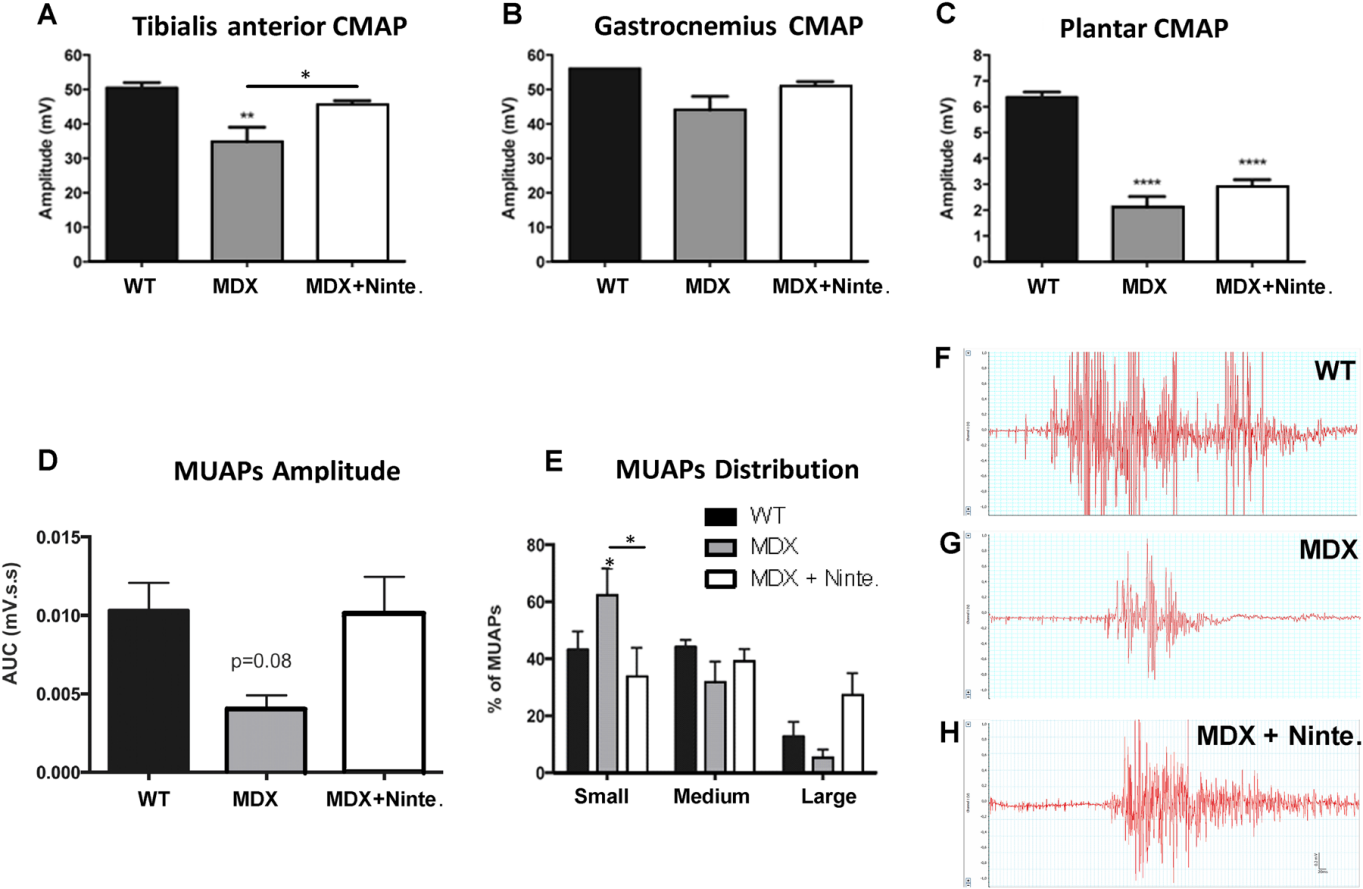
Motor nerve conduction tests and electromyographic analysis of WT, *mdx*, and nintedanib-treated *mdx* mice after 4 weeks of treatment. **a–c** Motor nerve conduction test showed that nintedanib administration preserved the compound muscle action potential (CMAP) i`n the tibialis anterior (**a**), gastrocnemius (**b**), and plantar (**c**) muscles. **d**, **e** Electromyography (EMG) of tibialis anterior. AUC quantification of electromyography (EMG) responses of the TA muscle after application of Von Frey filaments 5.47 to the ipsilateral hind paw (**d**). Percentage of MUAPs from each type that were activated (**e**) and EMG showed a lower proportion of small pathologic motor unit action potentials (MUAPs) in nintedanib-treated *mdx* mice. **f–h** Representative recordings of EMG bursts after nociceptive stimuli in WT (**f**), *mdx* (**g**), and nintedanib-treated *mdx* mice (**h**). Note that *mdx* animals showed lower amplitude and shorter duration MUAPs compared with WT and nintedanib-treated *mdx* mice. Data are expressed as means ± SD. Genetic background mouse strain C57BL (WT); *n* = 5, *mdx* mice (mdx), *n* = 5; nintedanib-treated *mdx* mice (mdx + Ninte.), *n* = 7. Data are expressed as means ± SD. *P < 0.05, **P < 0.01, **** *p* < 0.0001

We then used electrophysiological tests to analyze compound muscle action potential (CMAP) amplitude and morphology of the motor unit action potentials (MUAPs). We detected a statistically significant increase of the CMAP of the peroneus nerve registered at the tibialis anterior muscle in nintedanib-treated *mdx* mice compared with *mdx* mice (Fig. 2a). Moreover, we also found a non-significant tendency to an increase of CMAPs recorded in gastrocnemius (GM) and plantar interossei muscles (Fig. 2b, c). We did not identify changes in latency or conduction velocity. Electromyographic studies showed a significant change in the morphology of the MUAPs in the tibialis anterior muscle. WT muscles contained a similar proportion of small and medium MUAPs, whereas *mdx* mice had predominantly small MUAPs. The analysis of nintedanib-treated *mdx* mice revealed a significant decrease in the proportion of small MUAPs and an increase in the proportion of medium and large MUAPs, which was statistically significant compared with *mdx* mice (Fig. 2d–g).

### Nintedanib treatment reduces muscle fibrosis in mdx mice

**Fig. 3:**
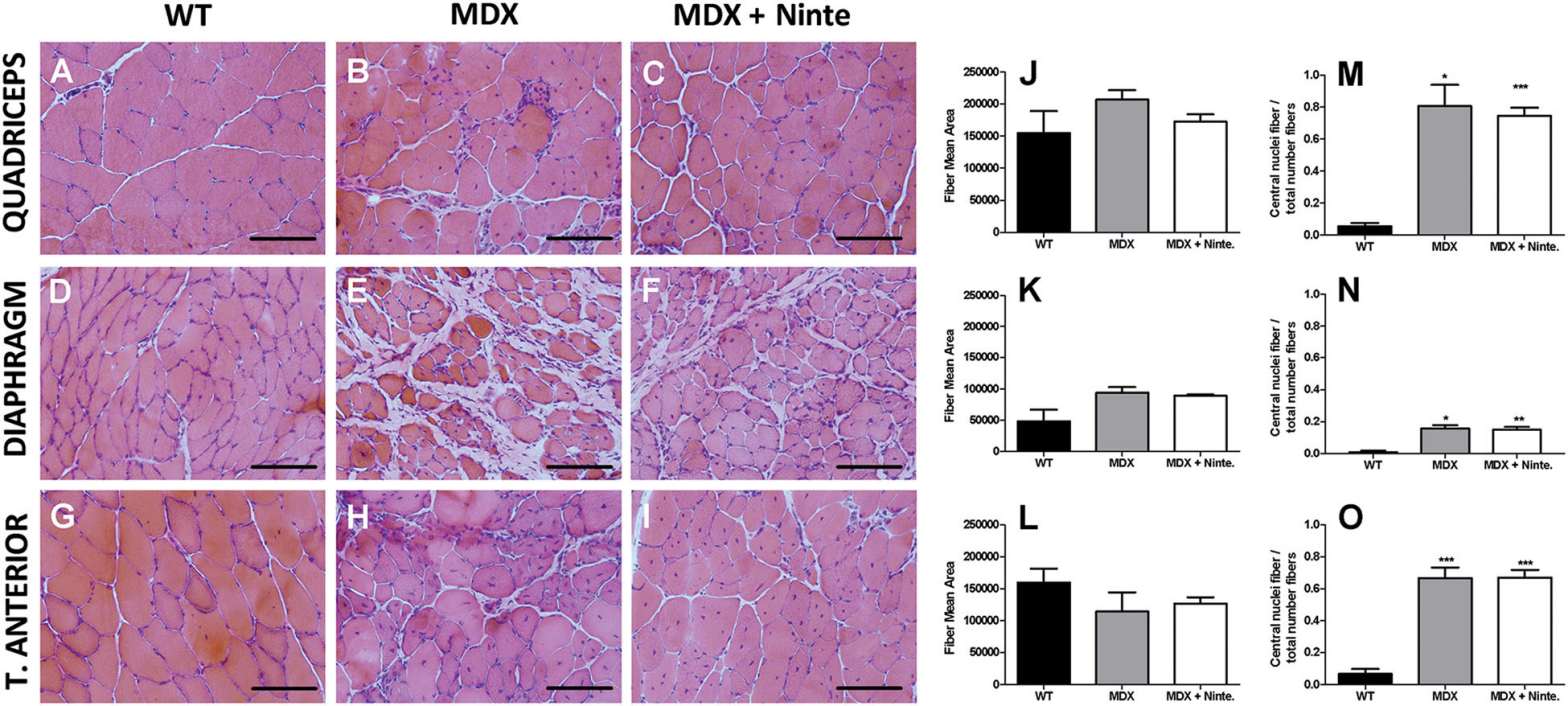
Histological analysis of muscle samples of WT, *mdx* and nintedanib-treated mdx mice. Hematoxylin and eosin (H&E) staining of muscle samples obtained (**a–i**). Representative examples of cross-sectional muscle fibers within quadriceps (**a–c**), diaphragm (**d–f**), and tibialis anterior (**g–i**) of WT, *mdx* and nintedanib-treated *mdx* mice. Schematic representation of the fiber mean area in quadriceps (**j**), diaphragm (**k**), and tibialis anterior (**l**) showed no differences on the size of the muscle fibers. Analysis of the proportion of central nucleated fibers in quadriceps (**m**), diaphragm (**n**), and tibialis anterior (**o**) did not present significant differences after 1 month of treatment with nintedanib (60 mg/kg). Data are expressed as means ± SD. Genetic background mouse strain C57BL (WT); *n* = 5, *mdx* mice (mdx), *n* = 5; nintedanib-treated *mdx* mice (mdx + Ninte), *n* = 7. Data are expressed as means ± SD. *P < 0.05, **P < 0.01 and ***P < 0.001. Scale bar = 100 µm

**Fig. 4:**
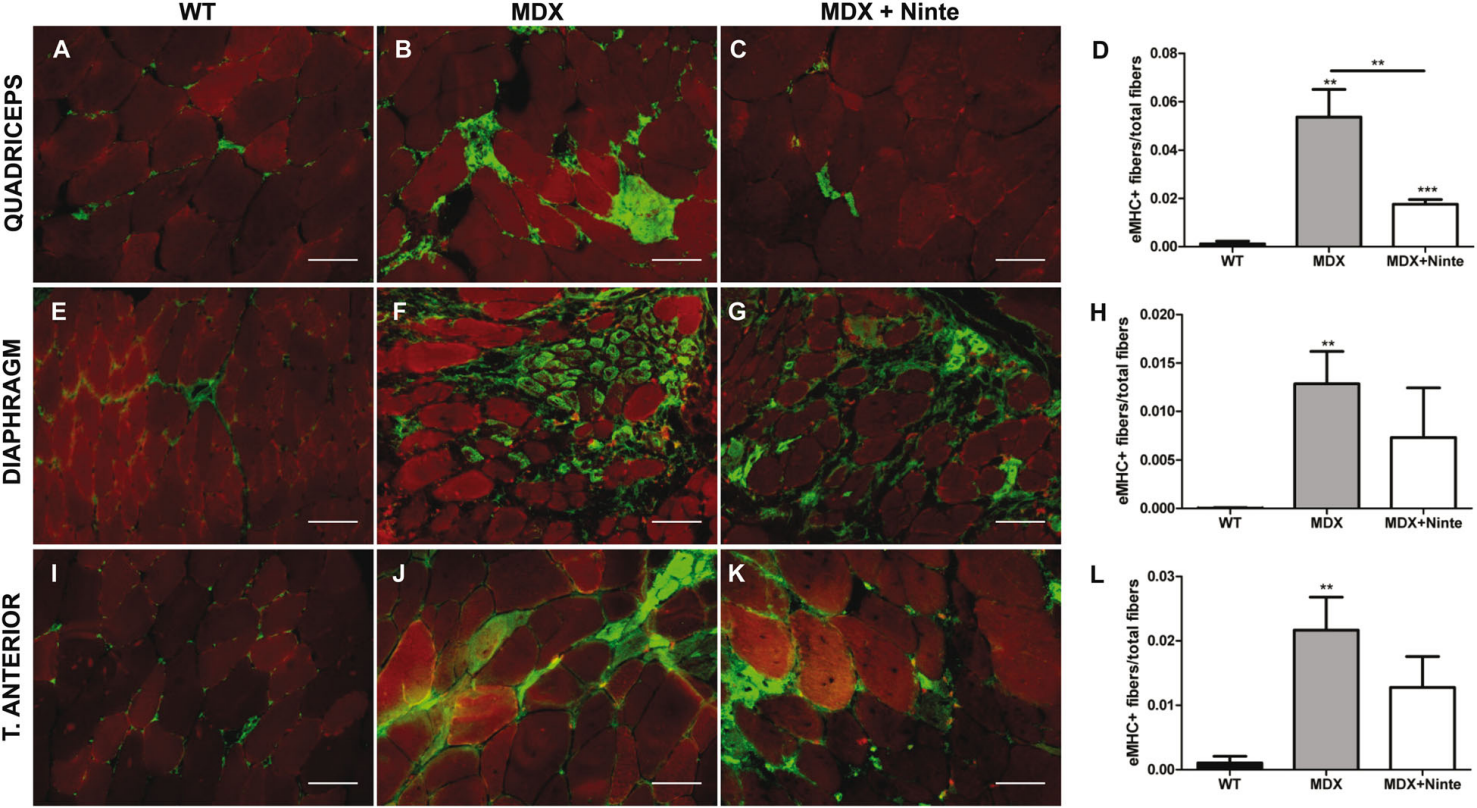
Nintedanib reduced the number of regenerative muscle fibers in *mdx* mice. Immunostaining of eMHC and quantification of positive fibers in muscle samples of WT, *mdx*and nintedanib-treated mdx mice (**a-l**). The number of eMHC-positive fibers (green) is reduced in quadriceps, diaphragm and tibialis anterior in *mdx* mice treated with nintedanib. Background in red is shown to localize the positivity within the muscle. Data are expressed as means ± SD. *P < 0.05 and **P < 0.01. Scale bar = 100 µm

**Fig. 5:**
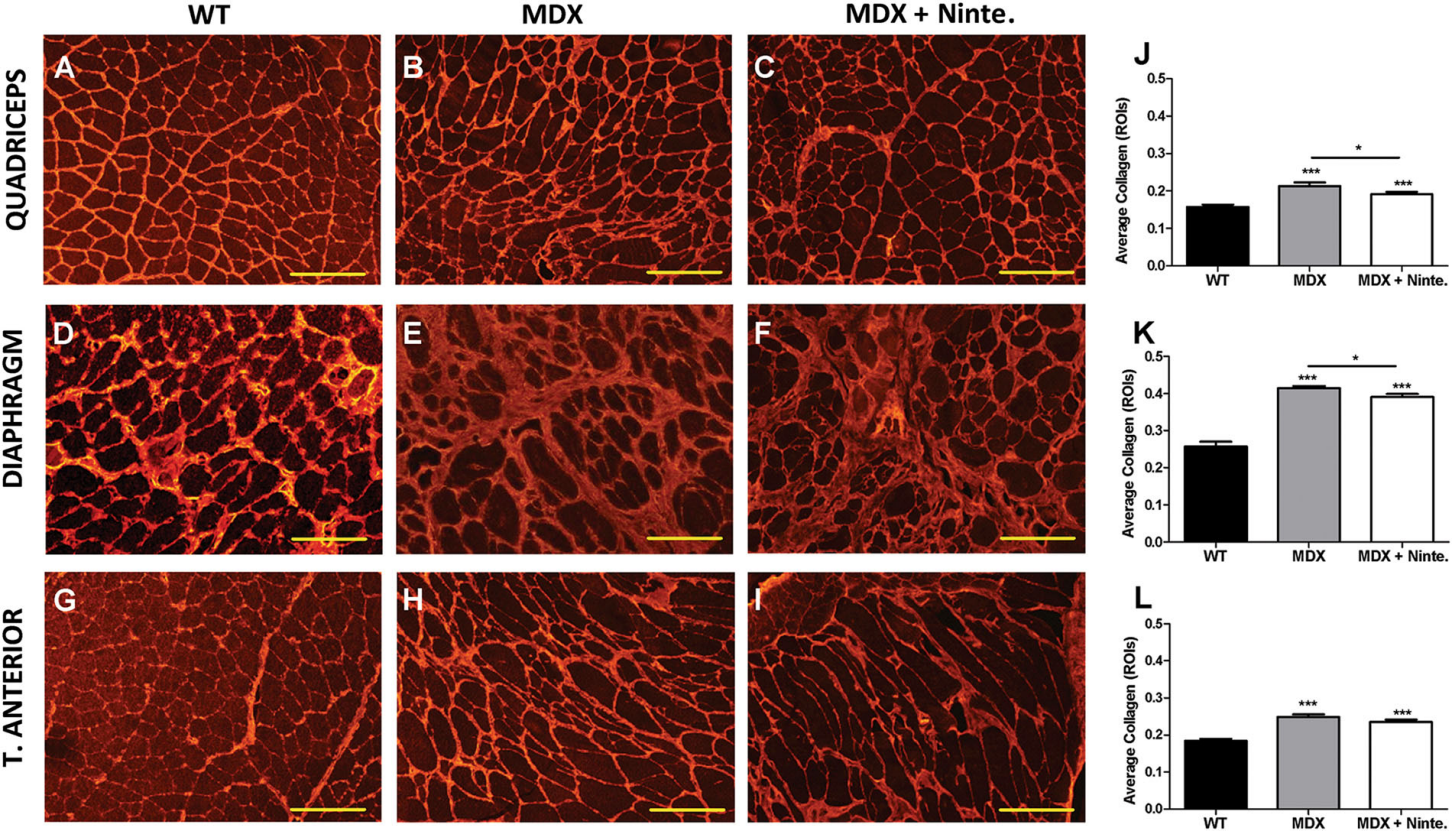
Nintedanib reduced skeletal muscle fibrosis in *mdx* mice. Representative pictures of immunofluorescence staining for collagen VI in quadriceps (**a–c**), diaphragm (**d–f**), and tibialis anterior (**g–i**) of WT, *mdx*, and nintedanib-treated *mdx* mice. Endomysial collagen deposition increased in all *mdx* muscles compared with WT group and decreased in nintedanib-treated *mdx* muscles, significantly in quadriceps (**j**) and diaphragm (**k**) but not in tibialis anterior (**l**) compared with non-treated *mdx* mice. Data are expressed as means ± SD. Genetic background mouse strain C57BL (WT); *n* = 5, *mdx* mice (mdx), *n* = 5; nintedanib-treated *mdx* mice (mdx + Ninte), *n* = 7. *P < 0.05. Scale bar of quadriceps and tibialis anterior = 200 µm. Scale bar of diaphragm *=* 100 µm.

The analysis of muscle histology after 1 month of treatment revealed significant differences between nintedanib-treated and non-treated *mdx* mice without any effect on apoptosis in the muscle of the animals (Fig. S5). We analyzed the diaphragm, which has been reported to be the most affected muscle in *mdx* mice^31^, the quadriceps and the tibialis anterior of WT, *mdx* mice, and nintedanib-treated *mdx* mice. Hematoxilin–eosin staining showed no differences either on the size of the muscle fibers or in the proportion of central nucleated fibers between *mdx* mice and nintedanib-treated *mdx* mice (Fig. 3). As nintedanib blocks the VEGF receptor, we also analyzed muscle vascularization. Although we did not find differences in the ratio of CD31-positive vessels per fiber among the three groups of mice, nintedanib treatment tended to reverse the increase observed in the ratio vessels/muscle fibers observed in the mdx mice (Fig.S6). To investigate in vivo the effect of nintedanib in the muscle, we quantified the number of eMHC-positive muscle fibers, which is an early marker of muscle regeneration. We observed a decrease in the number of muscle fibers expressing eMHC, in nintedanib-treated *mdx* mice compared with *mdx* mice (Fig. 4). We then analyzed the area of fibrotic tissue present in the skeletal muscles by quantifying collagen VI expression (Fig. 5). We observed a statistically significant reduction in the collagen VI area in the diaphragm (−5.6%), the quadriceps (−8.4%) and in the tibialis anterior muscles (−4.1%) of nintedanib-treated *mdx* mice compared with *mdx* mice (Fig. 5).

**Fig. 6:**
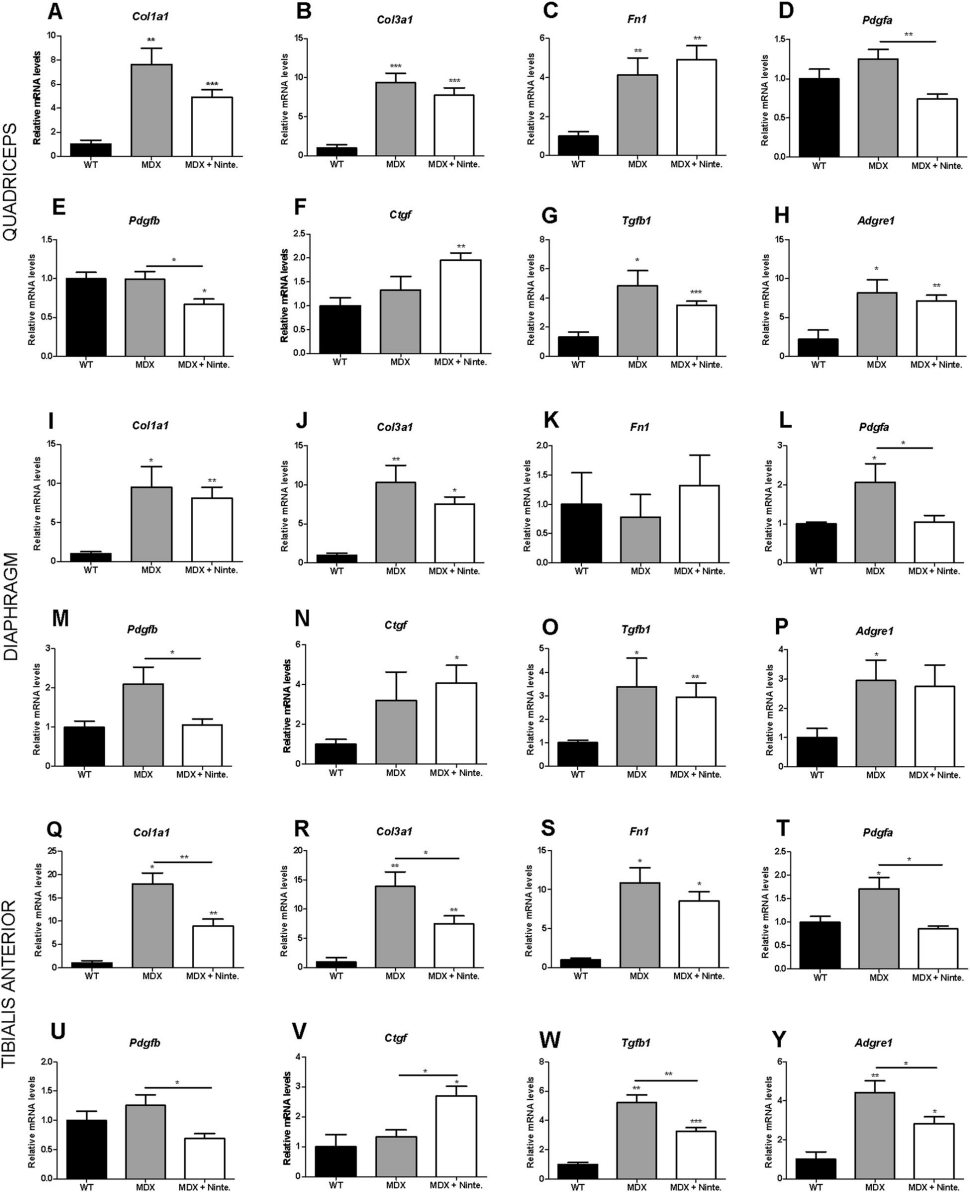
RT-qPCR studying mRNA expression of fibrosis-related genes. Collagen type I alpha 1 chain (Col1a1), Collagen type III alpha 1 chain (*Col3a1*), Fibronectin 1 (*Fn1*), Platelet-derived growth factor A (*Pdgfa*), Platelet-derived growth factor B (*Pdgfb*), Connective tissue growth factor (*Ctgf*), Transforming growth factor beta 1 (*Tgfβ1*), and Adhesion G protein-coupled receptor E1 (*Adgre1*) showed changes in relative abundance following nintedanib in quadriceps (**a–h**), diaphragm (**i–p**), and tibialis anterior (**q–y**). *Col1a1, Col3a1, Pdgfa, Tgfb1,* and *Adgre1* gene expression was increased in *mdx* mice compared with WT*. Col1a1, Col3a1, Pdgfa, Pdgfb, Tgfb1,* and *Adgre1* expression was reduced in all muscles analyzed from nintedanib-treated *mdx* mice compared with *mdx* mice. In contrast, *Ctgf* expression was increased in muscles from nintedanib-treated *mdx* mice compared with *mdx* mice. Data are expressed as means ± SD. Genetic background mouse strain C57BL (WT); *n* = 5, *mdx* mice (mdx), *n* = 5; nintedanib-treated *mdx* mice (mdx + Ninte), *n* = 7. *P < 0.05, **P < 0.01, ***P < 0.005

The mRNA expression of the extracellular matrix proteins *Col1a1, Col3a1*, and *Fn1*, of the growth factors *Pdgfa*, *Pdgfb*, *Ctgf,* and *Tgfb1*, and of *Adgre1* (Adhesion G protein-coupled receptor E1) encoding F4/80, a protein expressed by murine macrophage populations, were significantly increased or showed a tendency to increase in *mdx* mice compared with WT mice (Fig. 6). The mRNA expression of *Col1a1, Col3a, Tgfb1, Pdgfa*, and *Pdgfb* in quadriceps (Fig. 6d, e), diaphragm (Fig. 6l, m), and tibialis anterior muscles (Fig. 6t, u) of nintedanib-treated *mdx* mice was reduced compared with *mdx* mice. *Adgre1* mRNA expression was decreased by nintedanib in tibialis anterior and quadriceps muscles (Fig. 6o, r, w, y) from nintedanib-treated *mdx* mice compared with *mdx* mice. Surprisingly, nintedanib significantly increased *Ctgf* in the tibialis anterior muscles of *mdx* mice (Fig. 6v). A similar trend was also detected in the other muscles.

**Fig. 7:**
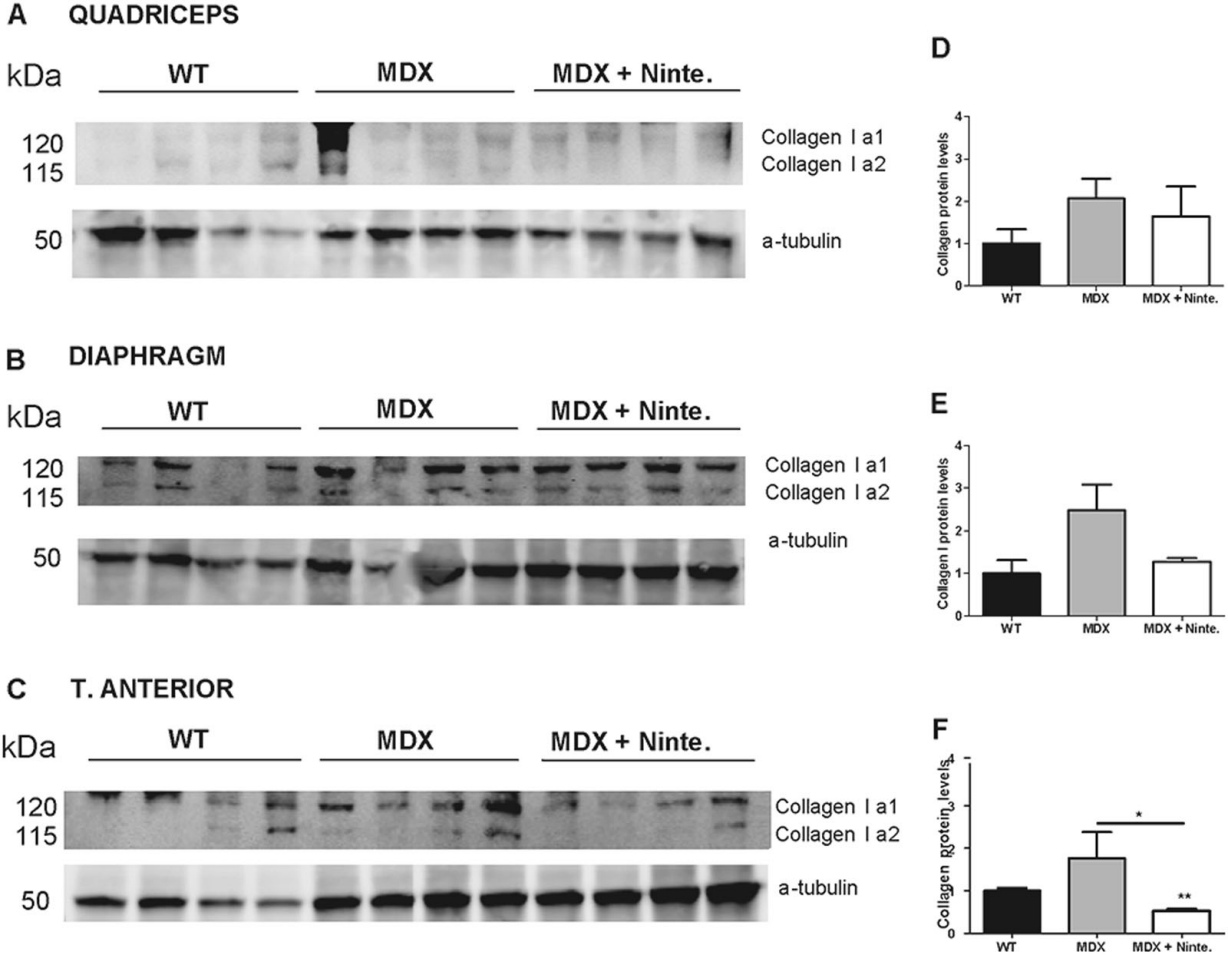
Nintedanib decreased collagen I expression in *mdx* muscles. Western blotting of Collagen I in quadriceps (**a**), diaphragm (**b**), and tibialis anterior (**c**) showed a reduction in the protein expression in diaphragm (**e**) and tibialis anterior (**f**) but not in quadriceps (**d**) of nintedanib-treated *mdx* mice compared with *mdx* mice by quantification of Collagen I normalized to α-tubulin levels. Data are expressed as means ± SD. Genetic background mouse strain C57BL (WT); *n* = 4, *mdx* mice (mdx), *n* = 4; nintedanib-treated *mdx* mice (mdx + Ninte), *n* = 4. *P ≤ 0.05 and **P ≤ 0.01

We confirmed the reduced expression *of Col1a1* and *Col3a1* by WB, with a significant decrease of collagen-1 in the tibialis anterior (*p* < 0.05) and a trend in the diaphragm (*p* = 0.08) of nintedanib-treated *mdx* mice compared with *mdx* mice (Fig. 7).

### Effect of nintedanib in heart fibrosis of the mdx mouse

We quantified the collagen VI and collagen III content in heart sections of WT, *mdx* mice, and nintedanib-treated *mdx* mice. We did not observe a significant difference in the area positive for collagen VI between WT and non-treated *mdx* mice (Fig. S7A–D). In contrast, collagen III was increased in *mdx* mice but it was not modified by nintedanib (Fig. S7E–H). However, gene expression showed a significant decrease in the *Col3a1* mRNA expression in nintedanib-treated *mdx* mice compared with non-treated mdx (Fig. S7J). mRNA expression of the *Col1a1, Fn1*, *Pdgfa*, *Pdgfb*, *Ctgf*, *Tgfb1*, and of *Adgre1* was also studied. We only observed a decrease in the expression of the *Adgre1* gene after treatment with nintedanib (Fig. S7I–P).

## Discussion

In the present study, we show that nintedanib reduces skeletal muscle fibrosis of 10 months old *mdx* mice after 4 weeks of treatment. The decrease in fibrotic tissue was associated with an improvement of muscle function evidenced by electrophysiological tests. In vitro experiments showed that nintedanib decreased fibroblasts proliferation and migration and reduced expression of fibrotic genes, supporting an anti-fibrotic effect of the drug.

Pharmacological approaches to treat muscle dystrophies aim to target the complex mechanism of fiber necrosis and fibrotic and fatty tissue deposition by restoring the proper expression of the mutated gene or by interfering with the pathological fibrotic cascade triggered by the lack of specific proteins^32^. Advances in the understanding of the fibrotic process have been recently achieved^33,34^. Continuous muscle fiber damage is associated with chronic infiltration of skeletal muscles by inflammatory cells, mainly macrophages. These cells release cytokines that contribute to the fibrotic process by activating fibro-adipogenic progenitor cells and fibroblasts resident in the skeletal muscle^22,35^. Among these cytokines, the growth factors of the PDGF family may have an important role. A series of evidences from the literature suggest that PDGF-AA has a profibrotic role in muscle dystrophies^35–39^. Treatment of *mdx* mice with imatinib, a TKI inhibiting v-Abl, c-kit, and PDGFRα, decreases muscle fibrosis^40^. The role of PDGF-BB is not so clearly established. Whereas it seems that PDGF-BB could have a role in the fibrosis observed in a number of tissues, several authors suggest that it can also play an important role in muscle regeneration in vivo^41,42^.

Nintedanib was shown to inhibit fibroblast proliferation and migration in vitro and to exert anti-fibrotic activity in different animal models of lung fibrosis^25,26^. We observed that muscle fibrosis was reduced in *mdx* mice after 4 weeks of treatment with nintedanib. This effect is probably explained by a decreased rate of fibroblasts proliferation, migration and by a reduction of the expression of different components of the extracellular matrix. Skeletal muscle fibrosis in DMD is caused by accumulation of different types of collagens, such as type I, III, and VI and also by fibronectin. We focused on collagen VI and III because they are the major components of the extracellular matrix and their levels correlates with muscle destruction^31,36,43,44^. We have observed a reduction in the expression of these extracellular matrix components both in vivo and in vitro. Moreover, we also observed a decrease in the expression of profibrotic growth factors such as PDGF-AA or TGF-β, which can certainly contribute to an attenuation of the fibrotic process in the treated mice. However, we observed an increase in CTGF levels in muscles of nintedanib-treated *mdx* mice, which could be explained by an enhanced fibrotic pathway not inhibited by our drug. This effect suggests that treatment with a combination of drugs blocking different pathways could have a more powerful anti-fibrotic effect. Using cell culture conditions with only 1% FBS, fibroblast proliferation was stimulated most prominently by PDGF-AA compared with VEGF or bFGF, which had no effect in those culture conditions. Hence, drugs targeting PDGFRα, the main receptor activated by PDGF-AA may be useful in the treatment of muscle dystrophies.

The preclinical investigation of new drugs to treat DMD is hampered by the lack of an animal model, which mimics the severity of muscle degeneration and natural history of the disease in patients. In the *mdx* mouse model we used, it has been shown that significant changes in muscles cannot be observed until 8–10 months of age^45^. Moreover, despite the presence of clear myopathic changes, mice do not present functional impairment until later in their life. Similar findings would apply to heart involvement. This might explain the absence of differences that we have observed in the Digigait test when comparing healthy C57 mice and non-treated *mdx* mice. Electrophysiology is an objective test that does not need the collaboration of the mice, providing valuable data about muscle integrity^46^. We observed significant changes in the amplitude of the CMAPs and in the morphology of the MUAPs using electrophysiological tests that were restored with treatment. These results are probably the consequence of early changes in the muscle structure produced by nintedanib that occur before clinical symptoms are observed.

The effect of specific TKIs, such as nintedanib, on the amount of fibrotic tissue present in the skeletal muscles of the patients may also improve muscle homeostasis. It is well known that muscle microenvironment is important for proper tissue regeneration^47^. In the process of muscle degeneration, there is an imbalance between proregenerative and prodegenerative processes. The increase in fibro-adipous tissue and the presence of chronic inflammation seem to impinge satellite cell proliferation, differentiation, and eventually muscle regeneration^48,49^. We hypothesize that nintedanib treatment could have a double benefit: (1) it reduces the amount of fibrotic tissue and (2) it could indirectly, restore the balance between proregenerative and prodegenerative processes. Our results support this hypothesis. We observed that nintedanib did not interfere with the differentiation ability of myoblasts in vitro or the resistance of myotubes to chemical damage. However, we did not see an increase in the number of regenerative muscle fibers in the animals treated suggesting that a reduction of the fibrotic process preserves skeletal muscle structure. Indeed, electromyograhy (EMG) studies showing restoration of MUAPs morphology indicate that muscle structure is more preserved in the treated animals supporting this hypothesis. It is important to take into account that we have observed a reduction of fibrotic tissue in an old animal, in which the fibrotic process has already developed. Treatment of younger animals for longer periods of time could result in a more sustained effect, although this needs to be confirmed in further experiments.

Different TKIs have been recently tested in murine models of muscle dystrophy. Imatinib has been tested in the *mdx* model producing a reduction in fibrotic tissue and in muscle fiber necrosis^21,40,50^. However, this drug, that is commonly used as a therapy for chronic myeloid leukemia and gastrointestinal tumors, is potentially associated with cardiac toxicity^51–53^. Nilotinib has been shown to reduce fibrosis in the *mdx* model through a mechanism related to the activation of apoptosis of FAP cells mediated by TGF-β ^43^. Nilotinib has been approved for the treatment of chronic myeloid leukemia and has been also tested in patients with dermal fibrosis^54,55^. Crenolanib is a TKI that inhibits selectively PDGFRα being tested in phase II clinical trials of gastrointestinal tumors^56^, melanomas^57^, or even acute myeloid leukemia^58^. This drug was tested in the *mdx* murine model and produced a reduction in collagen expression in the diaphragm of *mdx* mice^59^. Our promising results reinforce the potential role of specific TKIs such as nintedanib in the treatment of patients with muscle dystrophies. Nintedanib seems advantageous for a clinical exploration in DMD owing to its proven anti-fibrotic activity in patients with idiopathic pulmonary fibrosis, the fact that it has been approved for clinical use and its good safety profile^24,25^. However clinical experience is only available in patients with idiopathic pulmonary fibrosis, which are much older than patients with DMD. The potential impact of nintedanib as VEGFR inhibitor on bone and teeth formation during development in childhood is a safety concern that has to be carefully considered in a clinical testing scenario^60^.

In summary, nintedanib attenuated the fibrosis of skeletal muscles of *mdx* mice and induced a functional muscular improvement. The reduction of fibroblast proliferation and chemotaxis and the decreased expression of profibrotic genes by nintedanib may explain the mechanism of action of this drug. The data presented in this proof of concept study should be confirmed in larger preclinical studies before supporting a future test of nintedanib in a clinical trial in DMD patients. However, because of the lack of experience in young patients with nintendanib, a clinical trial to test safety, tolerability, and efficacy of the drug is certainly challenging.

## Material and methods

### Muscle biopsies

Muscle biopsies were obtained from patients who underwent orthopedic surgery. All participants signed an informed consent form and all the procedures were in accordance with the Helsinki Declaration. All in vitro experiments were performed in fibroblasts (proliferation, migration, and mRNA expression studies) and myoblasts (differentiation analysis) isolated from control muscle biopsies (*n* = 3).

### Isolation and culture of myoblasts and fibroblasts from human muscle biopsies

Human myoblasts and fibroblasts were isolated from muscle biopsies of three healthy muscles from control donors. In brief, muscle biopsies were minced into 1–2 mm pieces, transferred onto collagen type I (Sigma, St. Louis, MO)-coated dishes, and incubated in proliferation medium containing Dulbecco's Modified Eagle's Medium (DMEM) and M-199, in a 3:1 proportion, with 15% FBS, 2 mM l-glutamine (all from Lonza, Verviers, Belgium) (Lonza), 5 ng/ml basic fibroblast growth factor (Peprotech, Rocky Hill, NJ), and 1% penicillin–streptomycin (Lonza). After 5–7 days, cells started to sprout from the muscle explants. We isolated myoblasts using anti-CD56 coated microbeads (MiltenyiBiotec, BergischGladbch, Germany) and confirmed that the purity of the samples was higher than 95% performing immunofluorescence with antibodies against CD56 and desmin. Purified human myoblasts were cultured in proliferation medium. Fibroblasts were isolated from the CD56-negative fraction. We confirmed that > 95% of them were fibroblasts using anti-TE7 antibody (Millipore, Billerica, MA) by immunofluorescence. Fibroblasts were maintained in DMEM supplemented with 10% FBS, 2 mM glutamine, and 100 U/ml penicillin–streptomycin. All experiments performed with myoblasts and fibroblasts were repeated with cells isolated from all patients (*n* = 3) and were replicated in triplicate.

### Differentiation and repair assay of human myotubes

Myoblasts were seeded in gelatin-coated cover slips, grown until confluence in proliferation media and then cells were switched to low serum medium (2% of FBS) to induce differentiation in the presence of increasing doses of nintedanib (0.1, 0.2, 0.3, or 0.4 µM). In the repair assay, different doses of Nintedanib were added to untreated myotubes after 7 days of differentiation and incubated overnight and 2 h prior to the assay to ensure an effective inhibition of the TKI. After washing with Hank’s Balanced Salt Solution (HBSS) (Lonza) we added the injury solution (HBSS with 0.25 mM SDS (Sigma) and 1.8 mM CaCl_2_ (Sigma)) for 2 min. Control cells were treated with HBSS and 1.8 mM CaCl_2_ without SDS. Then, cells were washed with HBSS + 1.8 mM CaCl_2_ and incubated in recovery solution consisting of proliferation media at two time points: 90 s and 10 min. After that cells were exposed for 2 min to propidium iodide (Sigma) (20 µg/ml in HBSS) that stains nuclear DNA only if when the cell membrane is injured. Finally cells were washed with HBSS, fixed with 4% of paraformaldehyde (Sigma) in phosphate-buffered saline (PBS) and stained with Hoechst (100 µg/ml in PBS) (Invitrogen, Carlsbad, CA). The total number of nuclei and immunoprecipitation-positive nuclei were counted using Fiji software.

### Cell proliferation, viability, and cell death

To study the influence of nintedanib on cell proliferation, we seeded fibroblasts at 2000 cells/cm^2^ and cultured in growth medium containing 15% FBS. In these culture conditions, fibroblasts were treated daily with different concentrations of nintedanib (0.1, 0.2, 0.3, and 0.4 µM) (kindly provided by Boehringer-Ingelheim, Ingelheim, Biberach, Germany). To know which of the growth factors inhibited by nintedanib had a significant effect on fibroblast growth we analyzed cell proliferation on a medium that contained only 1% FBS. We added to the medium 10 ng/ml PDGF-AA (R&D Systems, Minneapolis, MN), 50 ng/ml VEGF-A (PeproTech, INC, Rocky Hill, NJ) or 10 ng/ml bFGF (PeproTech) with or without 0.4 µM nintedanib. Absolute cell numbers and viability, using LIVE/DEAD viability/cytoxicity kit (Invitrogen), were analyzed at days 2, 4, and 6 using MACS flow cytometer analyzer 10 (Miltenyi Biotech). Basal background was established using non-stained cells. To analyze cell death we performed a terminal deoxynucleotidyl transferase dUTP nick end labeling assay in fibroblasts treated with nintedanib and in muscle sections from the animal studies using in situ cell death detection kit (Sigma) following the manufacturer’s instructions. DNase I (Invitrogen) treatment was used as a positive control.

### Scratch and transwell assays

Migration of human fibroblasts was assessed using a scratch assay. In brief, human fibroblasts were seeded in proliferation medium until 90% confluence. In one set of experiments medium was changed to DMEM O/N, while in the other set, proliferation medium was maintained. The day after, cultures were scratched with a 0.2 ml pipette sterile tip to create a wound and washed in PBS and photographed. We assessed the effect of different doses of nintedanib (0.1, 0.2, 0.3, and 0.4 μM) on cell migration of proliferating fibroblasts after 24 h. In the set of experiments containing DMEM alone, nintedanib (0.4 μM) was incubated for 2 h. Then, PDGF-AA (20 ng/ml), TGFb (5 ng/ml), CTGF (55 ng/ml), bFGF 10 mg/ml, or VEGF (20 mg/ml) were added, incubated for 24 h, and photographed. Migration was quantified using Image J software. Inhibition of migration was expressed considering 100% as the amount of migration in the conditions without nintedanib. Three pictures of each replicate (*n* = 3) and each condition were taken using an inverted microscope (Olympus).

Transwell assays were performed in fibroblasts treated with or without 0.4 μM nintedanib for 2 h in a T75 flask. Then, cells were seeded into 8-μm pore-size transwell filters (Corning Incorporated, Corning, NY) at 4 × 10^4^ cells/well in 200 μl of OPTIMEM. In total, 600 μl of OPTIMEM with 10 ng/mL PDGF-AA or vehicle was added to the lower chamber. After 24 h of treatment at 37 °C, cells on the topside of the filter were removed by scrubbing twice with a cotton swab, and cells on the bottom side of the filter and cells in the well (migrating cells) were counted. Cells present in the insert membranes were fixed with methanol 70% for 10 min and stained for 30 min with Hoechst (Invitrogen). After three washes with PBS, filters were cut, mounted onto a slide, and observed with an Olympus fluorescence microscope. Chemotaxis toward Opti-MEM medium was considered as nonspecific chemotaxis.

### Fibroblast gene expression

To study fibroblasts mRNA expression, we seeded 3000 fibroblasts per cm^2^ and cultured them in growth medium. Fibroblasts were treated every 24 h with 0.4 μM nintedanib for 4 days and cell pellets were snap frozen with liquid nitrogen for the subsequent RNA processing.

### Mouse model

All animal procedures were performed according to the National Institutes of Health Guidelines for the Care and Use of Laboratory Animals and were approved by the Ethical Committee of the Universitat Autònoma de Barcelona. All mice used in the study were male and 10 months old at the start of the treatment period. Nintedanib was solubilized in sterile ultra-pure water (Braun, Rubi, Spain) and delivered to the animals using gavage. We treated seven C57BL/10ScSn-Dmdmdx mice with 60 mg/kg of nintedanib once daily for 4 weeks. As controls we used five untreated C57BL/10ScSn-Dmdmdx mice and five C57BL healthy mice.

After treatment, motor functional and electrophysiological studies were performed. Immediately after functional studies, the animals were euthanized and the diaphragm, tibialis anterior, quadriceps, and heart muscles were collected and frozen.

### Motor functional analysis: Digigait assay

Locomotion analysis was performed using the Digigait Imaging system (Mouse Specifics, Quincy, MA). In brief, digital video images of the underside of the mouse were collected with a high-speed video camera (80 frames/s) from below the transparent belt of a motorized treadmill. Each mouse was allowed to explore the treadmill compartment, with the motor speed set to zero, for 5 min. Then, the motor speed was set to 20 cm/s to collect the videos. A minimum of 200 images was collected for each walking mouse so that five to seven strides were captured in each run. Video images of 12.5 ms duration were digitized and the area (in pixels) of the paws was calculated with the DigiGait software.

### Motor nerve conduction studies

Motor nerve conduction studies were performed at 10 months of age in all the animals used in the study as previously described^61^. The sciatic nerve was percutaneously stimulated through a pair of needle electrodes placed at the sciatic notch, by means of single pulses of 0.02 ms duration (Grass S88). The CMAP was recorded from the tibialis anterior, plantar interossei, and GM muscles with microneedle electrodes^50^. All potentials were amplified and displayed on a digital oscilloscope (Tektronix 450 S) at settings appropriate to measure the amplitude from baseline to the maximal negative peak and the latency from stimulus to the onset of the first negative deflection, to the maximal negative peak and to the end of the wave. The recording needles were placed under microscope, guided by anatomical landmarks, to ensure reproducibility of needle location on all animals. During the tests, the mouse skin temperature was maintained between 34 and 36 °C using a thermostat heating pad. The observers were blinded to the experimental groups.

### Electromyography

Electromyography (EMG) recordings of MUAPs were obtained from the tibialis anterior muscle following a similar protocol to that previously described^62^. With the mice under anesthesia, EMG recordings were obtained in resting condition and following light noxious stimuli delivered to the ipsilateral paw to provoke bursts of EMG activity. Signals were digitized (Powerlab 6 T; ADInstruments) and fed into Chart software for post hoc analysis. MUAPs were then categorized into small, medium, and large amplitude as a representation of the three main types of motor units (S, FR, FF)^63^, and the amplitude and percentage of MUAPs from each class were measured. Von Frey (VF) monofilaments applied to the hind paw were also used to evoke bursts of EMG activity in the ipsilateral muscle. Since the force imposed by the filament is fairly constant, two different VF monofilaments were used to evoke responses, one with a known force of 10 g and 5.07 size and the other with a force of 26 g and a size of 5.46. The EMG responses recorded from the tibialis anterior muscle in response to those mechanical stimuli were recorded and analyzed to measure the area under the curve of each response using Chart software. At least, three different responses for each Von Frey filament were assessed per mouse.

### Immunostaining

Muscle samples were frozen in liquid nitrogen-cooled isopentane and serial 7 mm sections were cut with a Leica cryostat (Leica Microsystems, Wetzlar, Germany). For histological description diaphragm, tibialis anterior, quadriceps, and heart were stained with haematoxylin and eosin. Tissue sections were blocked with PBS containing 2% bovine serum albumin and fixed with acetone at room temperature for 10 min. After three washes with PBS, tissue sections were incubated with rabbit polyclonal anti-Collagen type VI (Millipore, Billerica, MA), rabbit anti-Collagen type III (Abcam, Cambridge, UK), mouse monoclonal anti-embryonic Myosin Heavy Chain (eMHC) (DSHB, Iowa City, IA), and rabbit polyclonal CD31 (Abcam). Appropriate Alexa-conjugated secondary antibodies were used. Fibroblasts from cell proliferation studies were stained with anti-Ki-67 (Abcam). In brief, cells were fixed in methanol, washed in PBS, and blocked with 5% goat serum for 1 h. Anti-Ki-67 (1/1000) was incubated for 1 h at room temperature (RT) and after washing steps with PBS, cells were incubated with goat anti-mouse Alexa594 for 1 h at RT. Positive cells were quantified compared with the total number of present cells. Pictures were taken with Olympus BX51 coupled to a DP72 camera. Five random pictures were taken per condition per triplicate.

### Collagen content quantification and muscle fibers geometric features extraction

Collagen content was labeled and was used to identify the contours of muscle fibers (darker regions). Aiming to avoid possible artefacts from the samples we took regions of interest (ROIs) with circular shape from each image. The ROIs were selected in regions where the tissue was not altered or broken. To calculate the collagen content in the images we took a maximum of three circular ROIs per image with a diameter of 700 pixels. An adaptive threshold was used to differentiate collagen and muscle fibers, binarizing each image depending of theirs levels of intensity.

To calculate geometric characteristics from the muscle fibers, we used an unique circular ROI with 2200 pixels of diameter. For this procedure, we selected 10 circular ROIs from each type of muscle for each category of mouse. The segmentation procedure and geometric features extraction was similar to the used in previous publications^64^. In this case, we only extracted eight geometrical features from the muscle fibers: mean area, standard deviation area, mean minor axis, mean major axis, mean relation between axis, standard deviation relation between axis, mean convex hull, and standard deviation convex hull. A manual correction step process was introduced to perfectly complete the identification of the fibers outlines. This was done using Adobe Photoshop CS6, followed by the final segmentation process. This final image was used to extract the eight geometric characteristics from all the cells into the ROI of each image.

### RNA extraction and reverse transcription

Total RNA was extracted from snap frozen fibroblast pellets and muscle samples of treated, non-treated *mdx* and control mice using RNeasy® Micro Kit (Qiagen, Hilden, Germany) and TRIzol (Invitrogen) respectively, following manufacturer’s instructions and stored at −80 °C. Contaminating DNA was digested with DNase I (invitrogen). RNA was quantified using a nanodrop ND-1000 spectrophotometer (Nanodrop Technologies Inc., Wilmington, DE, USA) and integrity was checked by 1% agarose gel electrophoresis. In all, 1 μg of total RNA was reverse-transcribed to complementary DNA (cDNA) using the High Capacity cDNA Reverse Transcription Kit (Applied Biosystems, Foster City, CA, USA).

### Real-time quantitative PCR (TaqMan) analysis

Real-Time PCR (qPCR) of cDNA obtained from cells and mouse tissues was performed using the TaqMan® Universal PCR Master Mix (Applied Biosystems, Foster City, CA, USA) and a 7900HT Fast Real-Time PCR System (Applied Biosystems, Foster City, CA). All mRNA-specific FAM-labeled primers/probe were purchased from Applied Biosystems and detected cDNA from the following genes: *Ctgf* (Mm01192933_g1), *Pdgfa* (Mm01205760_m1), *Pdgfb* (Mm00440677_m1), *Tgfb1* (Mm01178820_m1), *Col1a1* (Mm00801666_g1), *Col3a1* (Mm01254476_m1), *Fn1* (Mm01256744_m1), *Adgre1* (Mm00802529_m1), *PDGFA* (Hs00964426_m1), *CTGF* (Hs01026927_g1), *PDGFB* (Hs00966522_m1), *TGFB1* (Hs00998133_m1), *COL1A1* (Hs00164004_m1), *COL3A1* (Hs00943809_m1), *FN1* (Hs01549976_m1), *ACTA* (Hs00426835_g1), *MMP9* (Hs00957562_m1), *MMP2* (Hs01548727_m1), *MMP1* (Hs00899658_m1), *TIMP-1* (Hs99999139_m1), *TIMP-2* (Hs00234278_m1), *ADAM-12* (Hs01106101_m1), *ADAM-17* (Hs01041915_m1), *TP53* (Hs01034249_m1). All the experiments were performed in triplicate. Relative quantification was performed using the comparative Ct method and all results were compared with the control samples for each treatment after normalizing to an endogenous control (GAPDH (Hs99999905_m1) in the case of human fibroblasts and Tuba4a (Mm00849767_s1) in the case of mice using the Relative Quantification Manager software (Applied Biosystems, Foster City, CA). Data in bar graphs are presented as mean ± standard deviation of three independent samples in the case of fibroblasts, five in the case of C57 and *mdx* and seven in the case of treated *mdx* mice.

### Western blot

Total proteins from diaphragm, tibialis anterior, and quadriceps muscles were extracted in radioimmunoprecipitation assay lysis buffer (Sigma, St. Louis, MO) supplemented with 1% protease inhibitor cocktail (Roche, Indianapolis, IN) and 1% phosphatase inhibitor cocktail (Roche). The protein levels were measured using the bicinchoninic acid protein assay (Thermoscientific, Rockford, IL). Protein samples (50 μg) were denatured and separated on 10% sodium dodecyl sulfate polyacrylamide gel electrophoresis before transfer onto a polyvinylidene difluoride membrane and blocked with casein (Thermofisher, Rockford, IL). The blots were probed using different rabbit polyclonal anti-Collagen I antibodies (Abcam, Cambridge, UK). The blots were further incubated with secondary antibodies conjugated to fluorophores (LI-COR, Lincoln, Nebraska) and visualized using an Odyssey Imaging System (LI-COR). Protein levels were expressed relative to α-tubulin. Mouse monoclonal anti-α-tubulin antibody was purchased from Sigma (St. Louise, MO).

### Statistics

We used Student’s *t* test to compare quantitative measures between samples and analysis of variance to study repeated measures. Statistical significance was obtained at *p* < 0.05. Statistical studies were performed with SPSS® Statistics software version 21 from IBM® and graphics were developed using GraphPad Prism 5.01 software (La Jolla, CA).

## Electronic supplementary material

Supplemental figure 1

Supplemental figure 2

Supplemental figure 3

Supplemental figure 4

Supplemental figure 5

Supplemental figure 6

Supplemental figure 7

Figure legends of supplemental figures
