# Supplementary figures and images for: Nintedanib decreases muscle fibrosis and improves muscle function in a murine model of dystrophinopathy
Source: Cell Death Dis. 2018 Jul 10;9(7):776. doi: 10.1038/s41419-018-0792-6 (PMC6039566; doi:10.1038/s41419-018-0792-6)

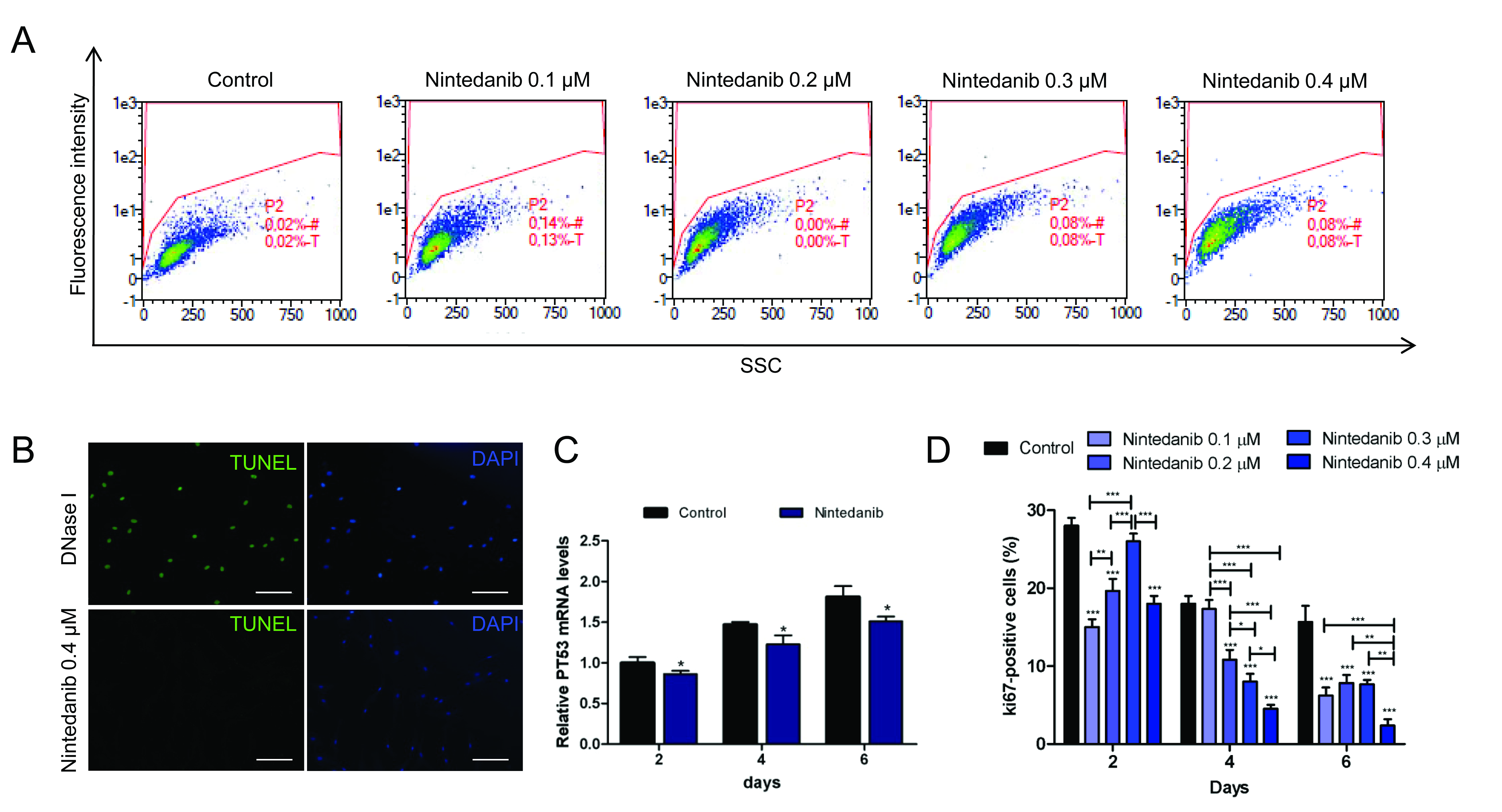

Supplement: Supplementary file 1 — Supplemental figure 1 [file 41419_2018_792_MOESM1_ESM.tif]

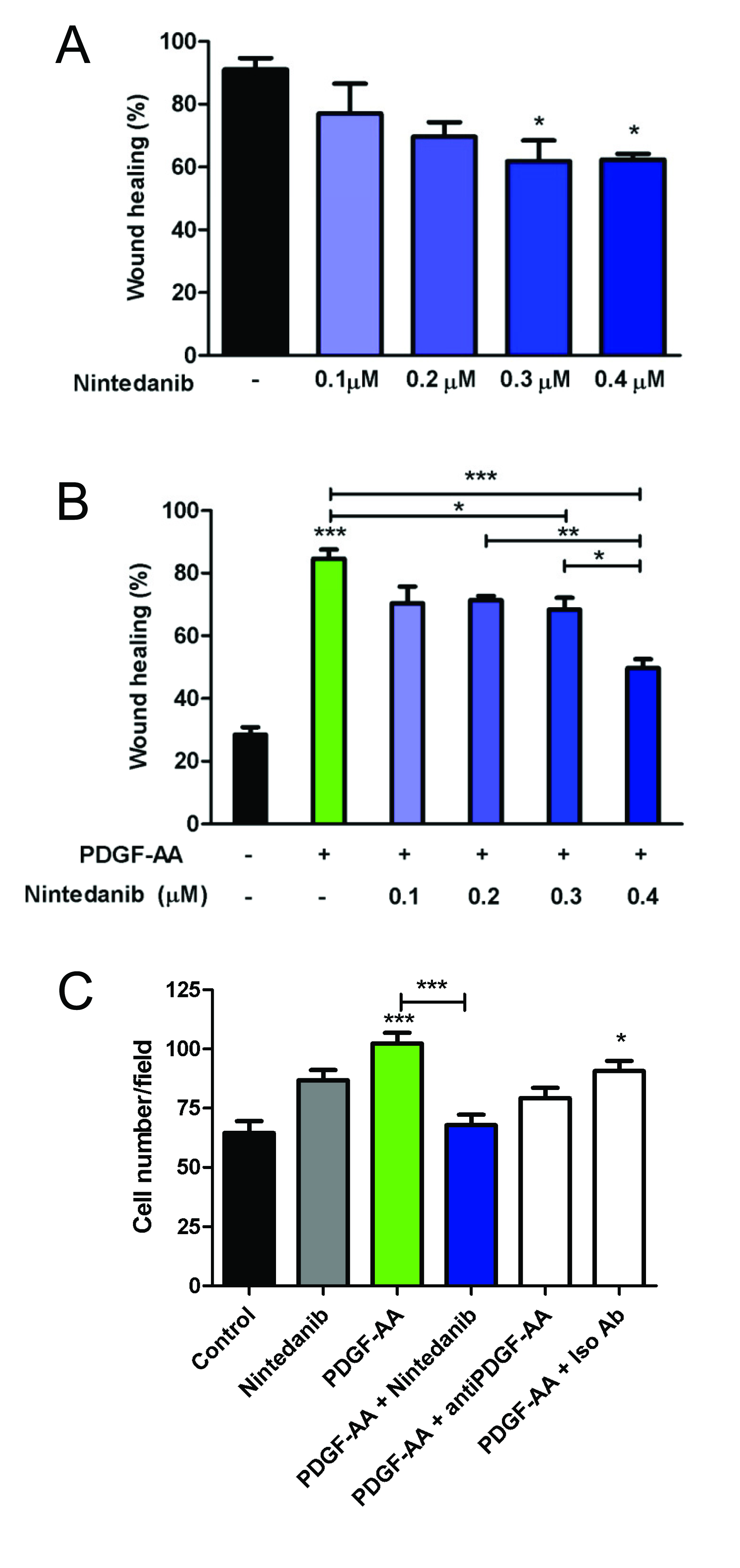

Supplement: Supplementary file 2 — Supplemental figure 2 [file 41419_2018_792_MOESM2_ESM.tif]

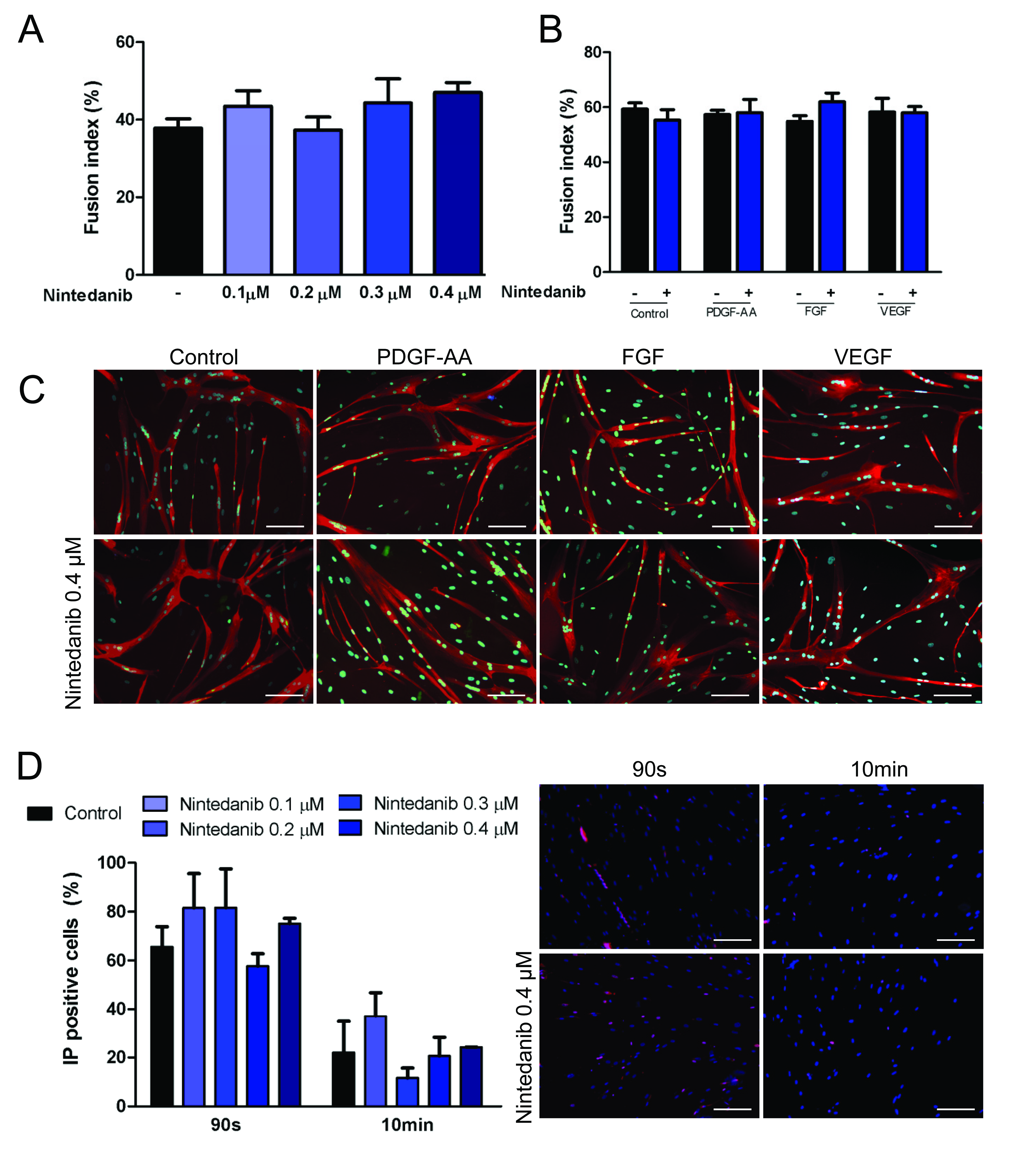

Supplement: Supplementary file 3 — Supplemental figure 3 [file 41419_2018_792_MOESM3_ESM.tif]

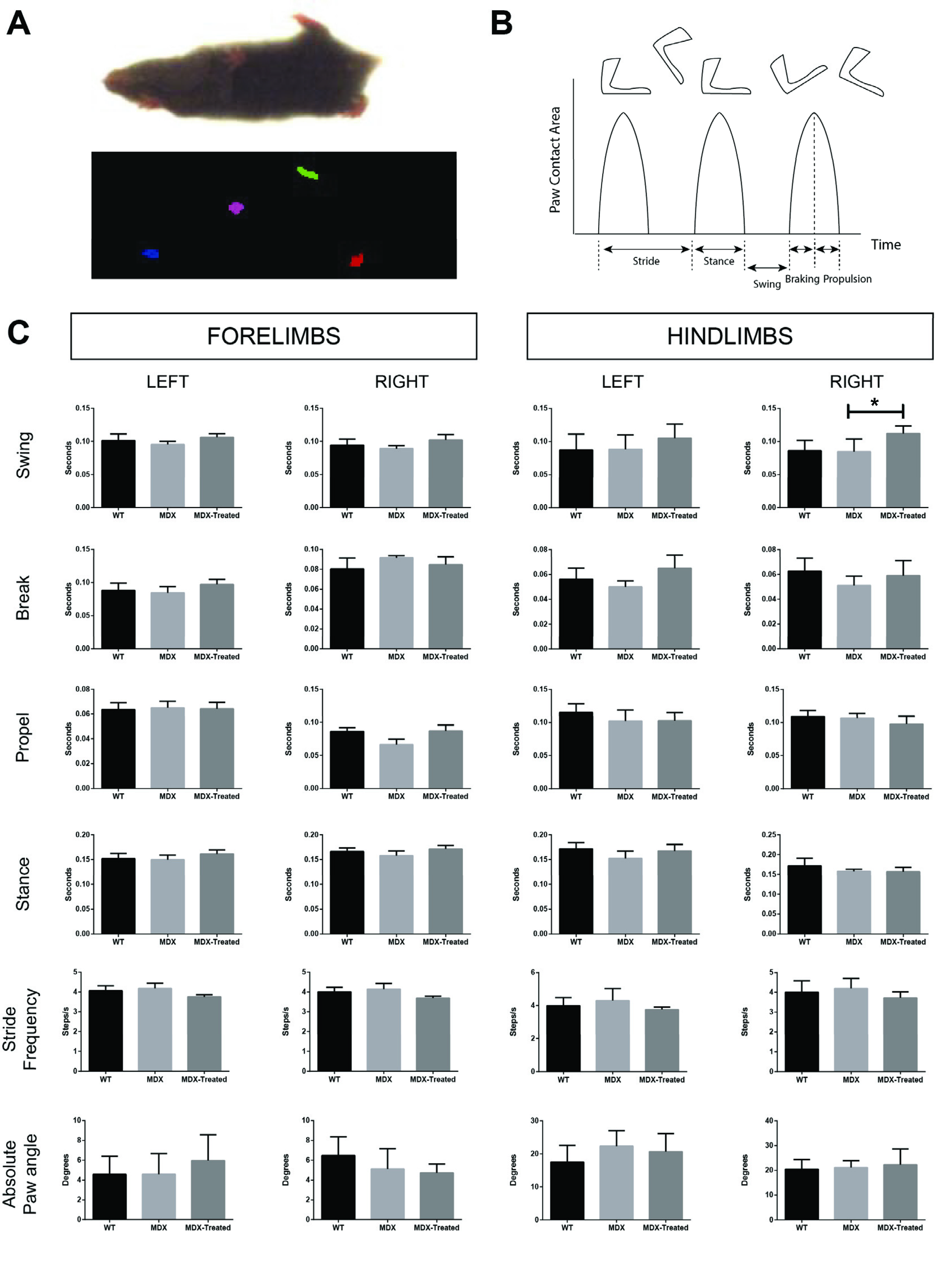

Supplement: Supplementary file 4 — Supplemental figure 4 [file 41419_2018_792_MOESM4_ESM.tif]

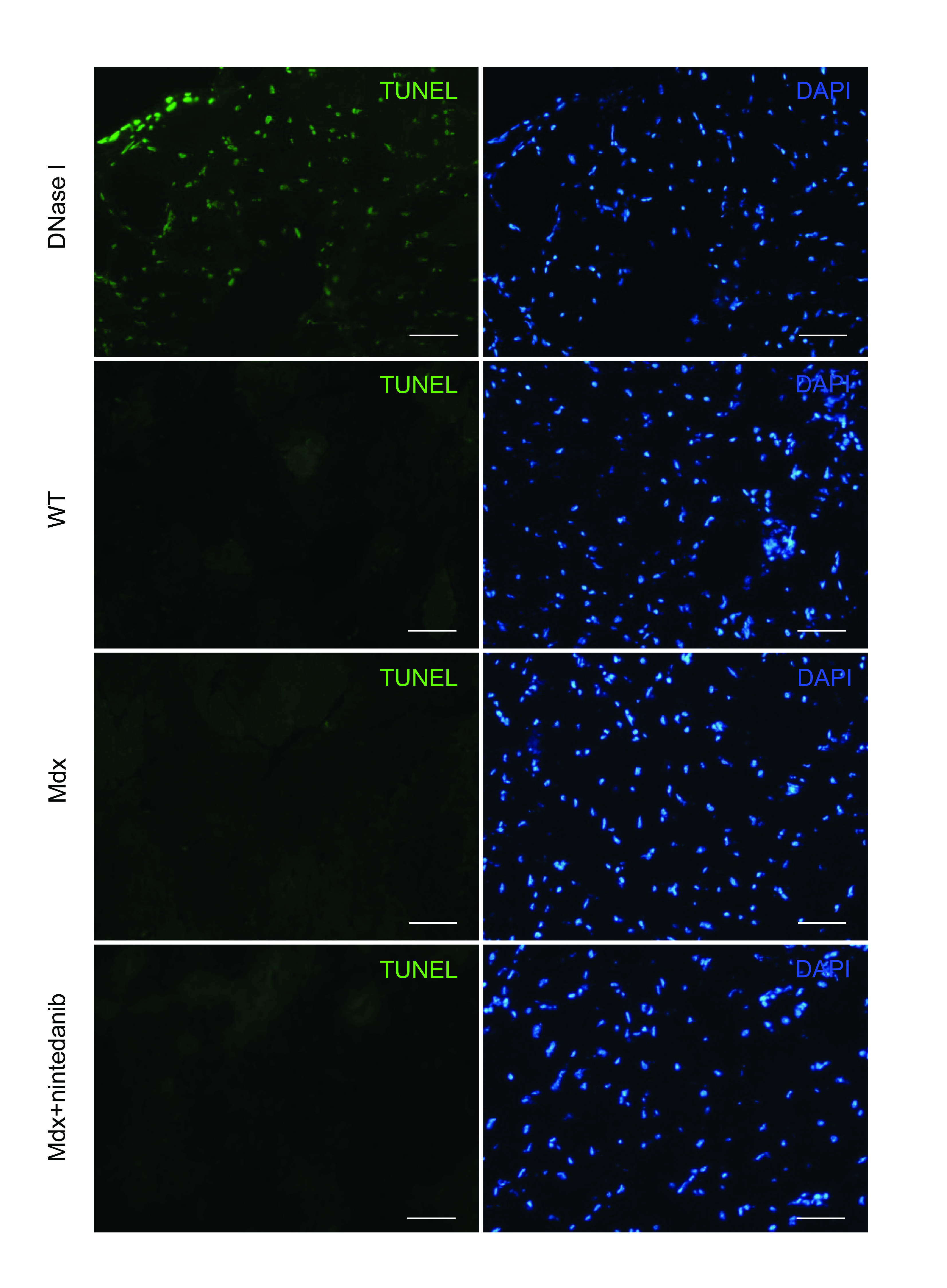

Supplement: Supplementary file 5 — Supplemental figure 5 [file 41419_2018_792_MOESM5_ESM.tif]

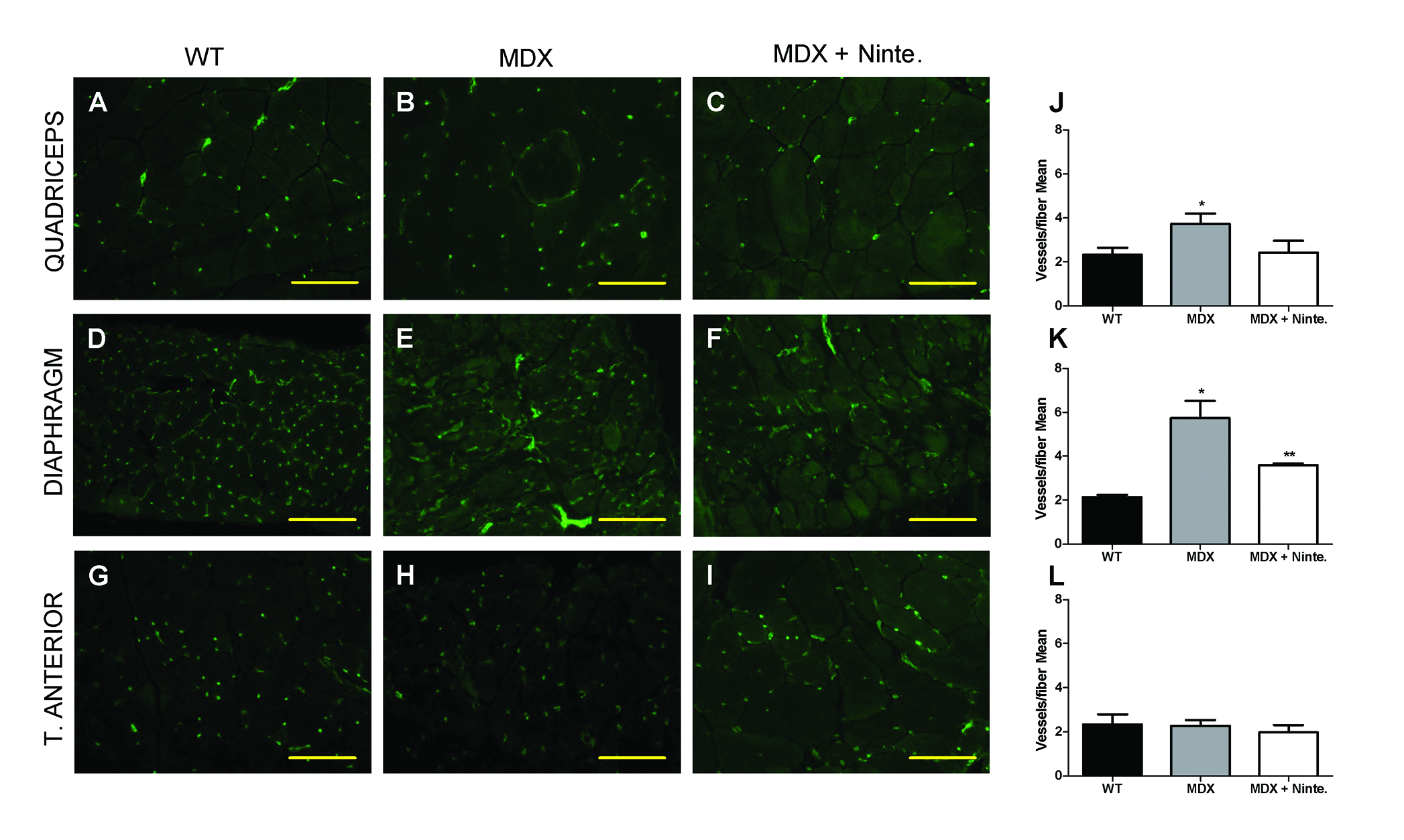

Supplement: Supplementary file 6 — Supplemental figure 6 [file 41419_2018_792_MOESM6_ESM.tif]

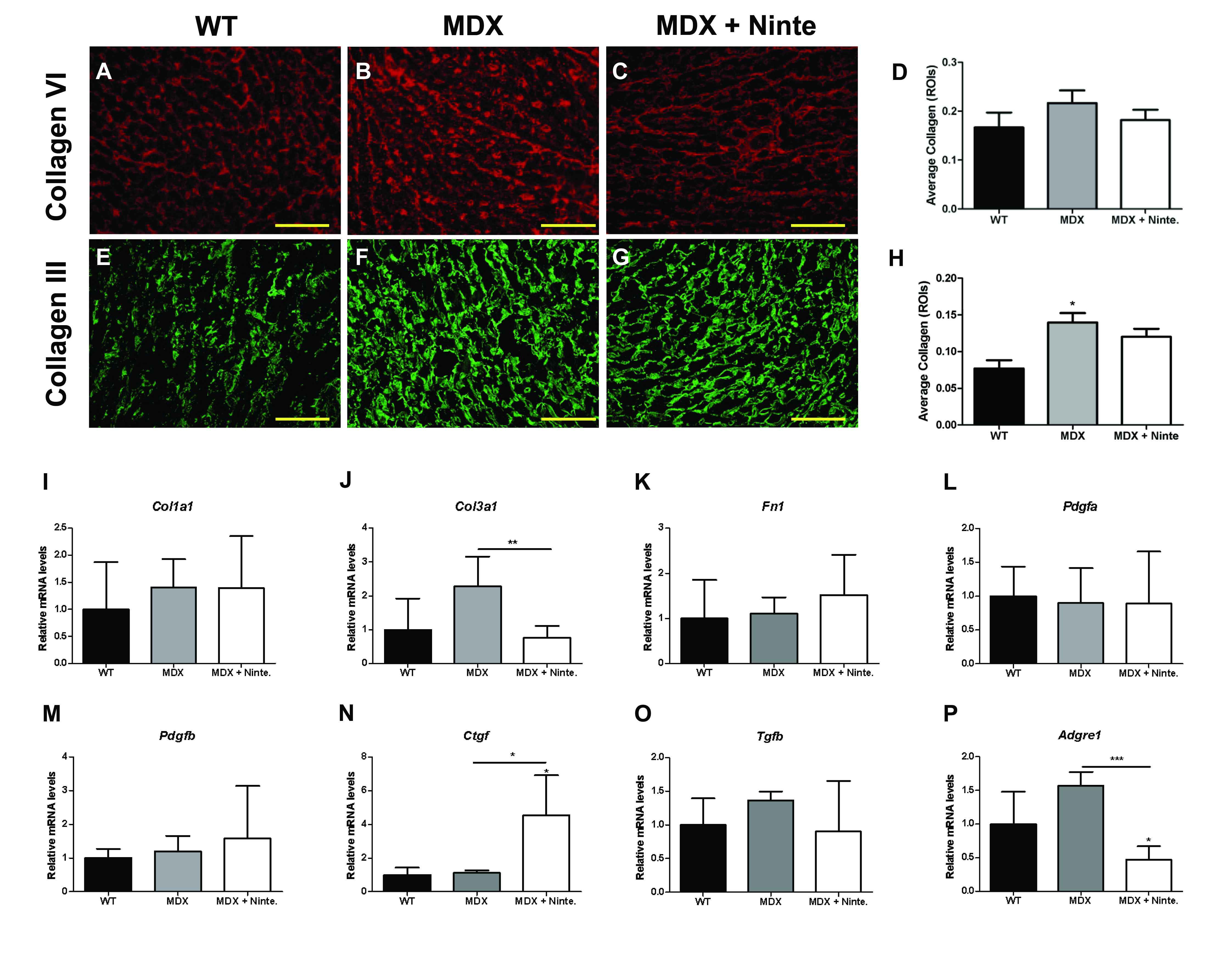

Supplement: Supplementary file 7 — Supplemental figure 7 [file 41419_2018_792_MOESM7_ESM.tif]
