# Supplementary material for: Nintedanib decreases muscle fibrosis and improves muscle function in a murine model of dystrophinopathy
Source: Cell Death Dis. 2018 Jul 10;9(7):776. doi: 10.1038/s41419-018-0792-6 (PMC6039566; doi:10.1038/s41419-018-0792-6)
Supplement: Supplementary file 8 — Figure legends of supplemental figures [file 41419_2018_792_MOESM8_ESM.docx]

Supplemental figures legends:

Figure S1. In vitro effects of nintedanid on proliferation and viability of fibroblasts. Treatment with nintedanib at increasing doses did not influence cell viability as analyzed using LIVE/DEATH viability kit with flow cytometry **(A)**. Nintedanib did not induced apoptosis of fibroblasts treated at 0.4 μM as analyzed using TUNEL assay **(B)**. Nintedanib at 0.4 μM significantly reduced PT53 expression by fibroblasts **(C).** The proportion of Ki67+ cells significantly decreased in a dose dependent manner **(D).** *P < 0.05, **P < 0.01, **** p<0.0001.

Figure S2. Effect of nintedanib on fibroblast migration and chemotaxis. Fibroblast migration was analyzed using scratch assay. Nintedanid treatment significantly decreased constitutive fibroblasts migration in a dose dependent manner **(A)** and reverted the promigratory effect of PDGF-AA on fibroblasts in a dose dependent manner **(B)**. Transwell migration assays to study chemotaxis showed that PDGF-AA at 10 ng/mL increased fibroblast migration, an effect reversed by nintedanib at 0.4 μM **(C)**. *P < 0.05, **P < 0.01, **** p<0.0001.

**Figure S3.** Effect of nintedanib on differentiation of myoblasts and repair of myotubes. Increasing doses of nintedanib did not influence differentiation of myoblasts in vitro measured by fusion index **(A)**. We did not observe any effect of PDGF-AA, FGFb and VEGF-A on myoblasts differentiation **(B)**. Representative pictures of myotubes cultured with PDGF-AA, FGF or VEGF with nintedanib are shown **(C),** Scale bar= 200 µm.. Repair assay after chemical damage induced by SDS at 0.25mM in myotubes cultured with increasing doses of nintedanib. The number of propidium iodide (IP) cells did not differ compared to control **(D)**, Scale bar= 100 µm.

Figure S4: Results of locomotion analysis (digigait). Digigait allows to study mouse locomotion analyzing digital video images of the underside (A). Different parameters can be studies such as stride, stance, swing, braking and propulsion (B). We did not observe significant differences in any of the parameters studied between wild type, *mdx* mice and nintedanib treated *mdx* mice. *P < 0.05.

Figure S5. Analysis of apoptosis on muscle biopsies from wild type, *mdx* mice and nintedanib treated *mdx* mice. Nintedanib treatment did not induced apoptosis in skeletal muscles of the treated mice analyzed using TUNEL assay. DNase I incubation of tissues were used as positive control. Scale bar= 100 µm.

Figure S6. Nintedanib did not influence muscle vascularization. **A-I:** Representative pictures of immunofluorescence staining with cluster of differentiation 31(CD31) antibodies in *quadriceps* (**A-C**), diaphragm (**D-F**) and *tibialis anterior* (**G-I**) of WT, *mdx* and nintedanib treated-*mdx* mice. **J-L:** quantification of vessels per fiber mean in *quadriceps* (**J**), diaphragm (**K**) and *tibialis anterior* (**L**). Data are expressed as means ± SD. Genetic background mouse strain C57BL (WT); n = 5, *mdx* mice (mdx), n = 5; nintedanib treated *mdx* mice (mdx + Ninte), n = 7. *P < 0.05, **P < 0.01. Scale bar *=* 100 µm.

Figure S7. Representative pictures and quantification of immunofluorescence staining for collagen VI **(A-D)** and collagen III in *heart* **(E-H)** of WT, *mdx* and nintedanib treated *mdx* mice. Data are expressed as means ± SD. Scale bar = 100 µm. RT-qPCR studying mRNA expression of fibrosis related genes in heart: Col1a1 (**I**), *Col3a1* (**J**), *Fn1* (**K**), *Pdgfa* (**L**), *Pdgfb* (**M**),*Ctgf* (**N**), *Tgfβ1* (**O**), and *Adgre1* (**P**). Data are expressed as means ± SD. Genetic background mouse strain C57BL (WT); n = 5, *mdx* mice (mdx), n = 5; nintedanib treated *mdx* mice (mdx + Ninte), n = 7. *P < 0.05, **P < 0.01, ***P < 0.005.
